# Marine flatworms as windows into the evolution of bilaterian neuropeptide signalling

**DOI:** 10.1111/jne.70259

**Published:** 2026-09-08

**Authors:** Ryo Nakamura, Mayuko Hamada, Xavier Bailly, Hirotaka Sakamoto, Tatsuya Sakamoto

**Affiliations:** ^1^ Ushimado Marine Institute, Faculty of Science Okayama University Okayama Japan; ^2^ Multicellular Marine Models Team CNRS – Sorbonne University – Station Biologique de Roscoff, Place Georges Teissier Roscoff France; ^3^ Department of Biology, Faculty of Environmental, Life, Natural Science and Technology Okayama University Okayama Japan

**Keywords:** Neuropeptide, Nervous system evolution, Platyhelminthes, Xenacoelomorpha

## Abstract

The evolutionary origin(s) of neuropeptide signalling is still largely unknown. It has been hypothesised that signal transmission via neuropeptides played a crucial role in the regulation of nervous systems in the bilaterian ancestor. Identification, expression and functional analyses of neuropeptides and their receptors across different taxonomic groups are important for better understanding the origin(s) of bilaterian neuropeptide signalling. An example is flatworms belonging to the Platyhelminthes (spiralian protostomes) and Xenacoelomorpha (sister to all Bilateria or sister to Ambulacraria). Despite their simple morphology (i.e., a body plan lacking a coelom and circulatory system), they often possess brain‐like central nervous systems and a number of bilaterian‐conserved neuropeptides. This feature makes them useful models for analyses of neuropeptides and their links to nervous systems. Although previous studies have revealed orthologous relationships between vertebrate neuropeptides and those found in both marine Platyhelminthes and Xenacoelomorpha, our work suggests possible ancestral functions of vasopressin/oxytocin (VP/OT) peptides (platytocin) in these groups.

## 1 INTRODUCTION

Neuropeptides are evolutionarily ancient signalling molecules that play essential roles in the regulation of physiology and behaviour, including feeding, metabolism, reproduction, stress responses and social behaviours^1^, ^2^, ^3^ across all major animal lineages. Remarkably, they are found not only in animals with nervous systems but also in neuron‐less animals such as placozoans, as well as choanoflagellates.^4^ This indicates that the neuropeptide signalling was already functioning before the emergence of neurons, highlighting their fundamental role in intercellular communication in animal evolution.^5^, ^6^, ^7^ Unlike classical fast‐acting neurotransmitters, which operate at precise synaptic junctions to elicit rapid and localised responses, neuropeptides are often released into the extracellular space and act via volume transmission. This mode of signalling enables them to modulate the activity of broader neural networks over longer distances and timescales.^6^

**FIGURE 1:**
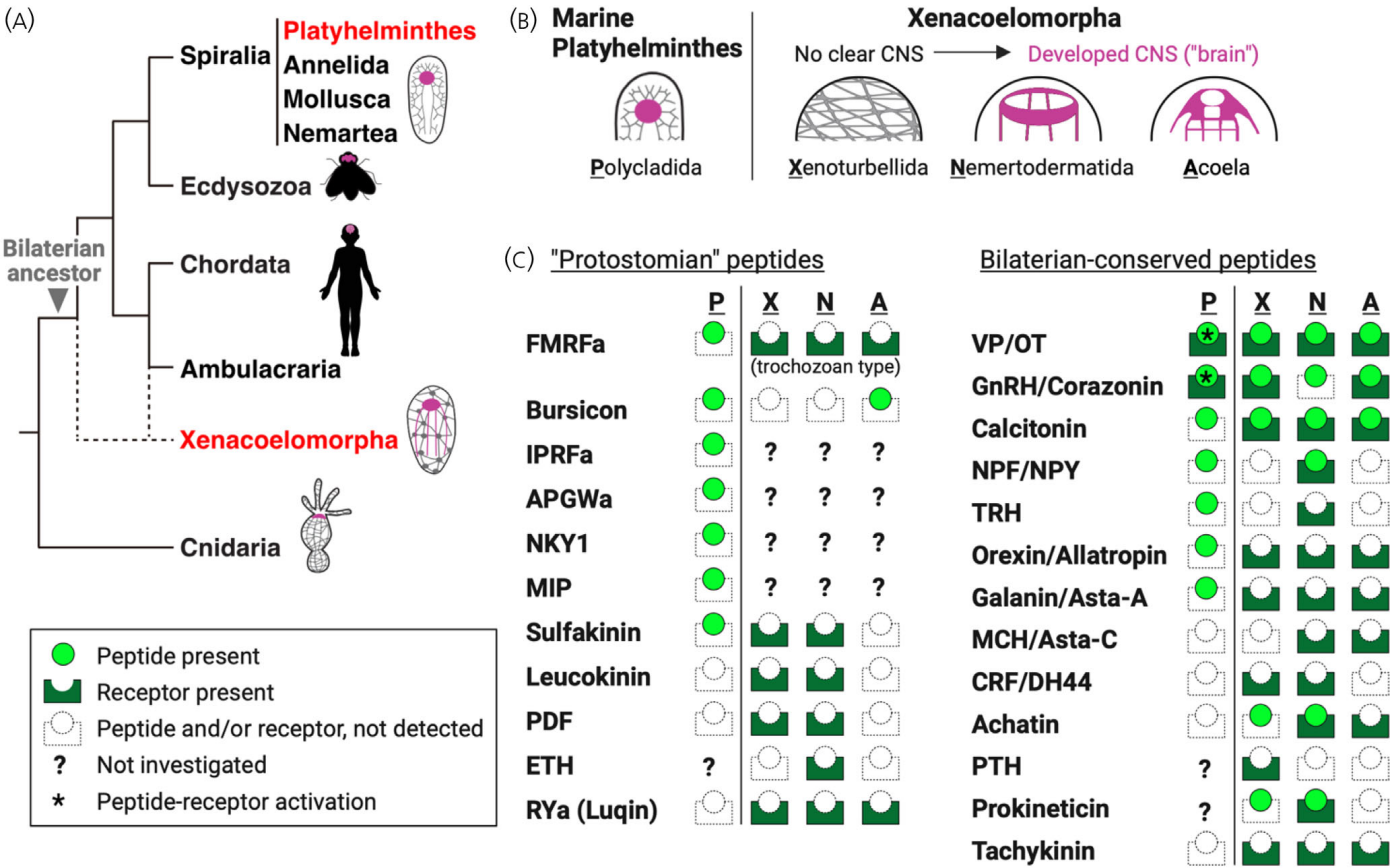
Comparison of nervous systems and neuropeptide repertoires between marine Platyhelminthes and Xenacoelomorpha. (A) Phylogenetic relationship in animal evolution. Dashed lines indicate alternative phylogenetic hypotheses. (B) Schemes of nervous system structures in the anterior region of Platyhelminthes (Polycladida) and Xenacoelomorpha, ranging from diffuse (Xenotubellida) to centralised (Acoela, Polycladida). Illustrations are modified from.^13^, ^19^, ^20^ (C) Identified neuropeptide repertoires of Platyhelminthes and Xenacoelomorpha show a mosaic pattern of conservation and lineage‐specific elements. The peptides and receptors are based on previous studies (Platyhelminthes,^19^, ^21^ Xenacoelomorpha^22^) and our current works. P, Polycladida; X, Xenoturbellida; N, Nemertodermatida; A, Acoela; FMRFa, FMRFamide; IPRFa, IPRFamide; APGWa, APGWamide; NKY, neuropeptide KY; MIP, myoinhibitory peptides; PDF, pigment dispersing factor; ETH, ecdysis triggering hormone; RYa, RYamide; VP/OT, vasopressin/oxytocin; GnRH, gonadotropin‐releasing hormone; NPF/NPY, neuropeptide F/neuropeptide Y; TRH, thyrotropin‐releasing hormone; Asta‐A, allatostatin‐A; MCH, melanin concentrating hormone; Asta‐C, allatostatin‐C; CRF, corticotropin‐releasing factor; DH, diuretic hormone; PTH, parathyroid hormone. Neural condensations/centralisations are shown in magenta in (A) and (B). The images were prepared using Biorender.com.

Specifically, the reorganisation/diversification of neuropeptide system repertoires in Bilateria is believed to be linked to the emergence of organised ‘brains’ by serving as integrated regulators of behaviour and homeostasis. Both Platyhelminthes and Xenacoelomorpha are flattened worms that are mostly in the millimetre‐size range. They are characterised by a sac‐like gut and the absence of a coelom, circulatory system and extracellular matrix surrounding the nervous system.^8^, ^9^ Despite such a simple anatomy, they often develop a central nervous system (CNS), which differs from the diffuse nerve nets, consisting of a central ganglion along with nerve cords in the anterior region.^10^, ^11^, ^12^, ^13^, ^14^ While Platyhelminthes are considered protostomes, the phylogenetic placement of Xenacoelomorpha is still debated. Its phylogenetically unstable position, sister to all remaining Bilateria (Nephrozoa) or sister to Ambulacraria (inside Deuterostomia), has led to its status as *incertae sedis* ^15^, ^16^, ^17^, ^18^ (Figure 1A). Furthermore, recent studies have even questioned the monophyly of deuterostomes.^23^ Regardless, Platyhelminthes and Xenacoelomorpha belong to distinct superphyla. Their simple anatomy and phylogenetic positions may make them unique models for understanding the principles of neuropeptide signalling repertoires and functions. In this review, we focus on neuropeptide signalling in Platyhelminthes and Xenacoelomorpha. By comparing these bilaterians, we might gain insights into the origin of neuropeptide signalling.

## 2 MARINE FLATWORMS, PLATYHELMINTHES

Among the protostomes, the study of neuropeptide systems has mostly been limited to several ecdysozoans (arthropods and nematodes)^24^, ^25^, ^26^ and spiralians (nemerteans, brachiopods, and phoronids),^27^ as well as molluscs and annelids.^3^, ^28^, ^29^, ^30^, ^31^, ^32^, ^33^, ^34^, ^35^, ^36^, ^37^, ^38^, ^39^ These studies have shown that they possess a relatively conserved neuropeptide complement with evidence for many ancestral bilaterian peptidergic systems.^27^, ^29^, ^31^

Platyhelminthes, other spiralians, may offer very simplified neural circuits and experimental tools for elucidating the functional roles of neuropeptides, but the neuropeptide complement has not been sampled thoroughly or was limited to single peptides. Studies that which have focused on freshwater planarians with substantial regenerative capacity and parasitic flatworms such as schistosomes and tapeworms failed to recover several ancestral neuropeptides from other protostomes. In contrast to most single‐cell RNA‐sequencing analyses of these freshwater planarians and parasitic flatworms,^40^, ^41^, ^42^ Piovani et al.^21^ presented a single‐cell atlas for larvae from the marine polyclad flatworm *Prostheceraeus crozieri*. They identified clusters of neurons primarily located in the apical organ (a possible larval CNS) characterised by the expression of both ‘protostomian’ peptides, such as IPRFamide, FMRFamide, XHFamide, FVRIamide, NKY1 (neuropeptide KY 1), LASGVamide, Bursicon, LSDWNamide, MIP (myoinhibitory peptides), 7B2, APGWamide and GNQQNxP, as in protostomes, and of bilaterian‐conserved peptides, such as GnRH/corazonin, calcitonin, neuropeptide Y/F (NPF/NPY), thyrotropin‐releasing hormone (TRH), allatostatin‐A/buccalin/kisspeptin/galanin and orexin/allatotropin, all of which show orthology also with vertebrate neuropeptides. While protostomian peptides have been acquired in a lineage‐specific manner, bilaterian‐conserved peptides are possibly more ancient, having appeared before the protostomian split, and may be linked to the emergence of the brain. Many freshwater planarians and parasitic flatworms have secondarily lost these peptides (Figure 1B).^43^

Sequencing the transcriptomes of other Polycladida (*Stylochoplana pusilla* and *Notoplana humilis*) enabled the first identification of vasopressin/oxytocin (VP/OT) molecules in Platyhelminthes,^19^ which we refer to as platytocin. We also identified its receptor, which is specifically activated by platytocin.^19^ Platytocin‐expressing cells and fibres are present in two pairs of neurons in the CNS, and platytocin functions as an ‘antidiuretic hormone’ while also coordinating a range of processes, including reproduction and chemosensory‐associated behaviour (also reported in vertebrates and other invertebrates).^44^, ^45^, ^46^, ^47^, ^48^, ^49^, ^50^, ^51^ The simplicity of polyclad flatworm systems may provide a unique advantage for understanding such complex processes. Unlike other (vertebrate) systems, which often feature numerous interconnected and overlapping pathways, the flatworm neural circuits are generally less complex.

Analysis of these transcriptome sequence data also revealed the presence of two GnRH/corazonins, a single GnRH‐type receptor and three corazonin‐type receptors. These peptides were tested as ligands for these receptors (our unpublished data). Our discovery of the distinct GnRH‐type and corazonin‐type signalling pathways in Polycladida may enable clear‐cut studies of these pathway functions and interactions within simpler neural circuits, uncovering the fundamental principles that can be applied to other, more intricate nervous systems (Figure 1C).

## 3 MARINE FLATWORMS, XENACOELOMORPHA

Xenacoelomorpha occupies a phylogenetically contentious position, either as sister to all remaining Bilateria (Nephrozoa) or as sister to Ambulacraria. Regardless of their exact phylogenetic placement, they represent a critical lineage for understanding bilaterian neuropeptide evolution. To date, the most comprehensive analysis of Xenacoelomorph neuropeptides has been conducted by Thiel et al.,^22^ who examined 13 species, including three clades of Xenacoelomorpha (two Xenoturbella, four Nemertodermatida and seven Acoela species), combining motif and sequence similarity searches, mass spectrometry and phylogenetic analyses. This study revealed the presence of both bilaterian‐conserved and ‘protostomian’ peptides in the phylum Xenacoelomorpha. Notably, nervous system organisation varies considerably within Xenacoelomorpha, from diffuse nerve nets in xenoturbella and nemertodermatids to more centralised at the anterior region in many acoels. Bilaterian‐conserved peptides (e.g., VP/OT, GnRH/corazonin, calcitonin, NPY/NPF, orexin/allatotropin, glycoprotein hormone‐related peptides, insulin‐like peptides and prokineticin‐like peptides) were present in one or two Xenacoelomorpha clades, but not in the Acoela – which possesses the most developed anterior CNS^11^, ^14^, ^20^, ^52^ (Figure 1B,C). Instead, Acoela exhibits Xenacoelomorpha‐specific multicopy peptides (MCPs) including four MCPs exclusive to Acoela, three shared between Acoela and Nemertodermatida, three unique to Nemertodermatida and one found in Xenoturbella. These findings suggest lineage‐specific organisation in their peptidergic signalling systems. However, our current work on the other acoel species, *Praesagittifera naikaiensis* (Acoela), has identified two bilaterian‐conserved peptides, GnRH and calcitonin, both expressed in the anterior region (possible ‘brain’), which is characterised by co‐staining for neural markers (e.g., GABAergic neurons, our unpublished data), indicating that some conserved bilaterian peptides have been retained in certain acoel lineages. This raises the possibility that neuropeptide repertoires of Xenacoelomorpha may be more conserved than expected. Immunostaining and transcriptomic analysis have detected ‘protostomian’ peptides, such as FMRFamide and MCPs (e.g., PxFVamide), showing similarities to those found in trochozoans (Spiralia).^20^, ^22^, ^53^, ^54^ This implies that ‘protostomian’ peptides may have existed in these bilaterian lineages. However, further confirmation is needed as these peptides have not yet been validated by LC–MS analyses in Xenacoelomorpha.

Single‐cell RNA sequencing of *Xenoturbella bocki* (Xenoturbella) and *Isodiametra pulchra* (Acoela) revealed that most neuropeptide‐producing or expressing cells are either neurons or secretory cells.^55^, ^56^ Interestingly, in *I. pulchra*, some peptides show broade, albeit lower levels of expression in non‐neuronal cells,^56^ suggesting a potential for versatile communication. This observation implies that neuropeptides in acoels are not strictly confined to the nervous system, possibly reflecting an ancestral feature or the evolution of a lineage‐specific peptidergic network in Acoela.

Taken together, peptidergic signalling in Xenacoelomorpha represents a mosaic of conserved and lineage‐specific elements (Figure 1C). In Acoela, the evolution of neural centralisation appears to have been accompanied by the diversification of novel peptides, while some conserved peptides were retained.

## 4 DISCUSSION

It is intriguing that bilaterian‐specific peptides such as VP/OT and GnRH/corazonin are conserved in both Platyhelminthes and Xenacoelomorpha (Figure 1C). This suggests that these peptides played important roles in physiological regulation prior to major bilaterian diversification. These findings support the hypothesis that a large‐scale repertoire reorganisation/diversification of neuropeptides and their receptors occurred during early bilaterian evolution.^6^ VP/OT and GnRH/corazonin are known to be involved in similar or specific physiological functions in Protostomia and Deuterostomia, respectively.^57^, ^58^ For example, the polyclad flatworm VP/OT‐like peptide, ‘platytocin’, exhibits multiple functions conserved across bilaterian phyla, suggesting that its role originated in the common ancestor of Bilateria. The VP/OT‐like peptide found in Xenacoelomorpha could be a key to reinforcing this scenario. Due to the growing understanding of the evolutionary conservation/diversification of neuropeptide functions in Bilateria, cross‐phylum comparisons using phylogenetically informative invertebrate models are required to elucidate the neuropeptide signalling mechanisms underlying complex behaviours and environmental adaptations.

The correlation between neuropeptide repertoires and CNS development is particularly fascinating. One of the Xenacoelomorpha clades, Acoela, despite possessing a developed CNS in xenacoelomorphs, appears to lack many of these conserved peptides and instead exhibits lineage‐specific neuropeptides. This pattern may support a previous hypothesis suggesting independent CNS evolution in Xenacoelomorpha.^59^ However, the evolutionary significance of peptide loss in Acoela remains unclear. The apparent absence of these peptides could reflect lineage‐specific adaptations or may simply result from incomplete investigation, as suggested by the detection of GnRH and calcitonin in the acoel *P. naikaiensis*. Further research is needed to clarify the evolutionary significance of neuropeptide repertoires across Acoela.

Advances in transcriptomics and peptidomics will be crucial in addressing these questions (see review by DeLaney et al.^60^). Future experimental studies should aim to combine neuropeptide gene knockouts or knockdowns with spatial expression profiling and neural circuit mapping (e.g., single‐cell transcriptomics, connectome analysis) using marine flatworms. This integrative approach, particularly when applied to the simple marine flatworms, will offer significant insights beyond their phyla and may improve our understanding of the evolution and functional diversification of neuropeptide repertoires and signalling in Bilateria.

## AUTHOR CONTRIBUTIONS

Conceptualization and writing original draft (R. N. and T. S.). Reviewing and editing (M. H., X. B. and H. S.).

## CONFLICT OF INTEREST STATEMENT

The authors declare no conflicts of interest.

## Data Availability

The unpublished data provided in the main text are available from the corresponding author upon request. Except for our unpublished data, all other data were obtained from recent publications.
